# Cesarean Scar Defect (Isthmocele) and Secondary Infertility: A Systematic Review of Reproductive Significance

**DOI:** 10.7759/cureus.115809

**Published:** 2026-09-05

**Authors:** Safaa Abdou El Sheshtawy, Shrein Farouk Okasha Elsayed, Samreen Oshiek Ahmed Shareef, Shaymaa Mohamed Assal, Asim Ahmed, Sara Awad Fageer Farah

**Affiliations:** 1 Obstetrics and Gynaecology, SEHA Corniche Hospital, Abu Dhabi, ARE; 2 Faculty of Medicine, Shendi University, Shendi, SDN; 3 Medicine and Surgery, University of Gezira, Wad Madani, SDN; 4 Obstetrics and Gynaecology, Tabarjal General Hospital, Tabarjal, SAU

**Keywords:** cesarean scar defect, isthmocele, reproductive outcomes, secondary infertility, uterine niche

## Abstract

Cesarean scar defect (CSD), also termed isthmocele or uterine niche, is an indentation or pouch-like defect in the myometrium at the site of a previous cesarean scar and may be associated with secondary infertility and adverse reproductive outcomes. This systematic review evaluated the reproductive significance of CSD across diagnostic, assisted-reproduction, surgical, and mechanistic evidence. PubMed/MEDLINE (Medical Literature Analysis and Retrieval System Online) and Scopus were searched from database inception through the final search date, supplemented by Google Scholar, PubMed Central, publisher webpages, DOI records, citation tracking, and manual reference-list screening. Original human studies examining the reproductive significance of CSD were eligible. Substantial clinical and methodological heterogeneity precluded meta-analysis; therefore, the findings were synthesized narratively. A total of 36 studies were included. Across the evidence base, the reproductive significance of CSD appeared greater when the defect was accompanied by adverse anatomical or functional features rather than by niche presence alone. These features included thin residual myometrium, intrauterine or intracavitary fluid, chronic endometrial inflammation, and altered uterine contractility. Some assisted reproductive technology (ART) cohorts reported lower implantation, clinical pregnancy, or live-birth outcomes among women with uterine niches. Surgical studies reported anatomical improvement and subsequent pregnancies or live births in selected patients. However, findings varied across patient populations, treatment approaches, and outcome definitions. CSD may be associated with secondary infertility and adverse reproductive outcomes, particularly when accompanied by adverse anatomical or functional features. The available evidence remains observational and heterogeneous, which limits causal inference and direct comparisons between treatment approaches. No single surgical approach was shown to be superior, and treatment selection should remain individualized and anatomy-guided. Future studies should use standardized CSD definitions and clearly defined fertility outcomes.

## Introduction and background

Cesarean delivery is one of the most frequently performed surgical procedures worldwide, and its increasing use has heightened interest in potential long-term maternal consequences [1,2]. One such consequence is cesarean scar defect (CSD), also termed isthmocele or uterine niche, which is generally defined as an indentation or pouch-like defect in the myometrium at the site of a previous cesarean scar [3]. Secondary infertility refers to difficulty achieving a subsequent pregnancy after at least one previous pregnancy. CSD has received increasing attention because of its association with abnormal uterine bleeding, pelvic symptoms, and possible adverse reproductive outcomes.

The reported prevalence of uterine niche varies considerably across studies, partly because of differences in diagnostic definitions, imaging modalities, timing of assessment, and study populations. Transvaginal ultrasonography and saline infusion sonography or sonohysterography are commonly used for diagnosis, with assessment typically including niche depth and width, residual myometrial thickness (RMT), and the presence of intrauterine or intracavitary fluid [3,4]. Although many niches may be incidental or asymptomatic, others are associated with postmenstrual spotting, abnormal uterine bleeding, pelvic pain, dysmenorrhea, dyspareunia, technical difficulty during embryo transfer, secondary infertility, and adverse reproductive outcomes [5,6]. Clinically, niche size, RMT, fluid accumulation, and associated symptoms may help distinguish an incidental anatomical finding from a defect that is potentially relevant to fertility.

The relationship between CSD and impaired fertility is multifactorial. Proposed pathways include retention of blood or mucus within the defect, persistent intrauterine fluid, local inflammation or chronic endometritis, altered endometrial receptivity, abnormal uterine contractility, and anatomical or technical difficulties during assisted reproduction [5-7]. These anatomical and functional abnormalities also provide a rationale for surgical correction in selected women with significant symptoms, infertility, or unfavorable scar characteristics. Surgical approaches include hysteroscopic, laparoscopic, robotic-assisted laparoscopic, vaginal, abdominal, and combined techniques. However, patient-selection criteria, RMT thresholds, choice of surgical approach, and the magnitude of reproductive benefit remain insufficiently standardized [7,8].

Previous systematic reviews have examined CSD mainly in relation to diagnosis, fertility, and surgical management [4,6,8,9], including a recent systematic review and meta-analysis evaluating isthmocele, surgical repair, and fertility outcomes [8]. However, clinically relevant evidence remains fragmented across scar anatomy, intrauterine fluid and inflammation, assisted reproduction, uterine function, and reproductive outcomes after repair. Bringing these findings together may help identify scar characteristics that are more likely to be relevant to infertility and may support closer evaluation or individualized management in selected patients. The present review therefore focuses on the broader reproductive significance of CSD rather than considering the anatomical defect, fertility outcomes, or surgical treatment in isolation.

The primary objective of this systematic review was to evaluate the evidence regarding the association between CSD/isthmocele and secondary infertility or adverse reproductive outcomes among women with previous cesarean delivery. Secondary objectives were to characterize fertility-relevant diagnostic and anatomical features, evaluate outcomes in assisted-reproduction settings and after surgical repair, and examine proposed mechanisms linking CSD with impaired implantation and fertility.

## Review

Methods

Reporting Framework and Protocol Status

This systematic review was conducted and reported in accordance with the Preferred Reporting Items for Systematic Reviews and Meta-Analyses (PRISMA) 2020 statement [10]. The review question, eligibility criteria, search strategy, study-selection process, data-extraction approach, risk-of-bias assessment methods, and synthesis plan were defined before final study selection and data extraction. This review was not prospectively registered in PROSPERO (International Prospective Register of Systematic Reviews), OSF (Open Science Framework), or another registry. No deviations occurred between the initial review design and the final study selection, data extraction, risk-of-bias assessment, outcome interpretation, or narrative synthesis. The final literature search was conducted on July 10, 2026.

Review Question and Eligibility Framework

The review question was: among women of reproductive age with a history of cesarean delivery, what evidence is available regarding the association between CSD, isthmocele, uterine niche, or cesarean scar diverticulum and secondary infertility or reproductive outcomes, and what diagnostic, anatomical, assisted reproductive technology, surgical, and pathophysiological findings may explain this association?

The PICOS (Population, Intervention, Comparison, Outcomes, Study design) framework used to define eligibility is summarized in Table 1. 

**Table 1:** PICOS framework used to define review eligibility ART: assisted reproductive technology; CSD: cesarean scar defect; ICSI: intracytoplasmic sperm injection; IVF: in vitro fertilization; PICOS: population, intervention or exposure, comparator, outcomes, and study design.

PICOS element	Definition used in this review
Population	Reproductive-age women with a previous cesarean delivery, including women with secondary infertility, fertility desire, symptomatic cesarean scar defect, or ART/IVF/ICSI treatment when relevant subgroup data were reported separately.
Exposure or intervention	Cesarean scar defect, isthmocele, uterine niche, cesarean scar diverticulum, cesarean scar syndrome, or surgical repair using hysteroscopic, laparoscopic, robotic-assisted laparoscopic, vaginal, abdominal, or combined approaches.
Comparator	Women without a niche or cesarean scar defect, preoperative status, expectant management, alternative repair methods, alternative ART strategies, or no comparator in eligible single-arm studies and case series.
Outcomes	Secondary infertility, spontaneous conception, pregnancy, clinical pregnancy, live birth, miscarriage, implantation and ART/IVF/ICSI outcomes, intracavitary or intrauterine fluid, residual myometrial thickness, niche dimensions, uterine contractility, chronic inflammation, chronic endometritis, microbiome or mycobiome findings, and symptom or anatomical improvement relevant to fertility.
Study designs	Original human cohort, case-control, cross-sectional, prospective or retrospective observational, non-randomized surgical or interventional studies, and case series with extractable fertility, reproductive, ART, diagnostic, anatomical, surgical, or mechanistic outcomes.

Eligibility Criteria

Studies were eligible if they included women with a previous cesarean delivery and evaluated CSD, isthmocele, uterine niche, cesarean scar diverticulum, or cesarean scar syndrome in relation to fertility or reproductive outcomes. In this review, these terms were treated as equivalent when they referred to a defect or niche at the site of a previous cesarean incision. Eligible studies could also report assisted reproductive technology (ART) outcomes, diagnostic or anatomical findings, outcomes after surgical repair, or plausible fertility-related mechanisms. Studies involving broader populations were included only when relevant CSD or fertility subgroup data were reported separately.

Studies were excluded if they involved an ineligible population, did not assess CSD or an equivalent scar-niche condition, lacked a relevant fertility, reproductive, ART, diagnostic, anatomical, surgical, or mechanistic outcome, or provided insufficient extractable information. Reviews and other non-original reports, protocols without completed results, conference abstracts without full-text reports, animal or laboratory-only studies, and single-case reports without extractable group-level data were excluded.

Reports that focused exclusively on cesarean scar pregnancy were excluded unless they provided eligible information on isthmocele, infertility, or broader reproductive outcomes. For duplicate or overlapping reports, the most complete peer-reviewed report with the most relevant extractable data was retained. Reports that could not be assessed reliably in English were excluded.

Reports for which the full text could not be retrieved were recorded separately as reports not retrieved and were not included in the full-text eligibility assessment. Studies were not excluded on the basis of methodological quality or risk-of-bias assessment. Risk-of-bias assessment was conducted after study inclusion and was used to inform interpretation of the evidence rather than to determine eligibility.

Information Sources and Search Strategy

A systematic literature search was conducted in PubMed/MEDLINE and Scopus from database inception to July 10, 2026, without a publication-date restriction. Embase and the Cochrane Central Register of Controlled Trials were not searched. The strategy was adapted to the indexing structure and field syntax of each database. Search concepts covered CSD-related terminology, fertility and pregnancy outcomes, ART and embryo transfer, intrauterine fluid and inflammatory features, uterine function, and surgical repair.

Records retrieved from PubMed/MEDLINE (Medical Literature Analysis and Retrieval System Online) and Scopus were exported into a standardized spreadsheet and consolidated. Duplicate checking was based on title, authors, publication year, digital object identifier (DOI), journal, and publisher information. The combined database searches identified 506 records. After removal of 71 duplicate records, 435 unique database records remained.

Supplementary literature discovery used Google Scholar, PubMed Central, publisher webpages, DOI records, backward and forward citation tracking, and manual screening of relevant reference lists. These sources were used to identify potentially eligible reports rather than as formal bibliographic databases. Supplementary records were verified against bibliographic or full-text sources before eligibility assessment. Supplementary searching identified 11 additional records, resulting in a total of 446 records available for title and abstract screening across database and supplementary sources. Only completed English-language full-text reports containing sufficient extractable information were eligible. Database-specific search strategies are summarized in Table 2. 

**Table 2:** Database-specific search strategies DOI: digital object identifier; ICSI: intracytoplasmic sperm injection; IVF: in vitro fertilization; TITLE-ABS-KEY: title, abstract, and keywords.

Database	Search period and fields	Search strategy
PubMed/MEDLINE	Database inception to July 10, 2026; title/abstract	(isthmocele[Title/Abstract] OR "cesarean scar defect"[Title/Abstract] OR "caesarean scar defect"[Title/Abstract] OR "cesarean scar niche"[Title/Abstract] OR "caesarean scar niche"[Title/Abstract] OR "uterine niche"[Title/Abstract] OR "cesarean scar diverticulum"[Title/Abstract] OR "caesarean scar diverticulum"[Title/Abstract] OR "uterine scar defect"[Title/Abstract] OR "cesarean section scar defect"[Title/Abstract] OR "caesarean section scar defect"[Title/Abstract]) AND (infertil*[Title/Abstract] OR subfertil*[Title/Abstract] OR fertil*[Title/Abstract] OR pregnan*[Title/Abstract] OR conception[Title/Abstract] OR implantation[Title/Abstract] OR miscarriage[Title/Abstract] OR "live birth"[Title/Abstract] OR IVF[Title/Abstract] OR ICSI[Title/Abstract] OR "embryo transfer"[Title/Abstract] OR hydrometra[Title/Abstract] OR "intrauterine fluid"[Title/Abstract] OR inflammation[Title/Abstract] OR endometritis[Title/Abstract] OR endometriosis[Title/Abstract] OR peristalsis[Title/Abstract] OR hysteroscop*[Title/Abstract] OR laparoscop*[Title/Abstract] OR "vaginal repair"[Title/Abstract] OR "robotic repair"[Title/Abstract] OR isthmoplasty[Title/Abstract])
Scopus	Database inception to July 10, 2026; title, abstract, and keywords	TITLE-ABS-KEY(isthmocele OR "cesarean scar defect" OR "caesarean scar defect" OR "cesarean scar niche" OR "caesarean scar niche" OR "uterine niche" OR "cesarean scar diverticulum" OR "caesarean scar diverticulum" OR "uterine scar defect" OR "cesarean section scar defect" OR "caesarean section scar defect") AND TITLE-ABS-KEY(infertil* OR subfertil* OR fertil* OR pregnan* OR conception OR implantation OR miscarriage OR "live birth" OR IVF OR ICSI OR "embryo transfer" OR hydrometra OR "intrauterine fluid" OR inflammation OR endometritis OR endometriosis OR peristalsis OR hysteroscop* OR laparoscop* OR "vaginal repair" OR "robotic repair" OR isthmoplasty)

Study Selection

All identified records were consolidated in a standardized spreadsheet, and duplicates were removed before screening. Potentially overlapping reports were checked at full text, with the most complete peer-reviewed report retained when necessary. Study selection was conducted in two stages. Two reviewers independently screened titles and abstracts against the eligibility criteria. Records considered potentially eligible by either reviewer proceeded to full-text assessment. The same reviewers then independently assessed the available full-text reports. Disagreements were resolved through discussion and consensus. A third reviewer adjudicated disagreements that remained unresolved. Reports that could not be obtained were recorded as not retrieved. A specific reason for exclusion was documented for every report assessed at the full-text stage, using categories corresponding to the prespecified eligibility criteria. A formal inter-reviewer agreement statistic was not calculated.

Data Extraction

Two reviewers independently extracted data from each included study using a standardized Excel extraction sheet (Microsoft Corporation, Redmond, Washington, United States). The extraction form was piloted on a small sample of included studies and refined before final extraction. Extracted variables were grouped into study characteristics (author, year, country, design, setting, sample size or analytical denominator, and population); diagnostic and anatomical features (diagnostic method, niche definition, RMT, niche dimensions, and intrauterine or intracavitary fluid); exposure or intervention, comparator, and follow-up; and fertility, pregnancy, ART, surgical, mechanistic, and adverse outcomes when reported. The completed extraction records were compared for completeness and consistency. Disagreements were resolved through discussion and consensus, with third-reviewer adjudication available when required. Study authors were not contacted to obtain additional or missing information.

Outcomes

The primary outcomes were secondary infertility; pregnancy-related outcomes, including spontaneous conception, pregnancy, clinical pregnancy, live birth, and miscarriage; and implantation or ART/in vitro fertilization (IVF)/intracytoplasmic sperm injection (ICSI) outcomes associated with CSD or isthmocele. Secondary outcomes included diagnostic and anatomical characteristics, such as RMT, niche depth, width or volume, intrauterine or intracavitary fluid, and hysteroscopic, sonographic, or hysterosalpingographic findings. Additional secondary outcomes included symptom and anatomical improvement after surgical repair and mechanistic findings involving chronic inflammation, chronic endometritis, altered uterine contractility, microbiome or mycobiome changes, pelvic adhesions, tubal factors, endometriosis, and other plausible implantation-related pathways. Differences in outcome definitions, measurement methods, assessment timing, analytical denominators, and follow-up duration were considered during synthesis.

Risk-of-Bias Assessment

Two reviewers independently assessed risk of bias using appraisal tools selected according to study design. Observational cohort and case-control studies primarily examining exposure-outcome associations were evaluated using the Newcastle-Ottawa Scale (NOS) [11]. Analytical cross-sectional studies were assessed using the JBI Critical Appraisal Checklist for Analytical Cross-Sectional Studies, while case series were assessed using the JBI Critical Appraisal Checklist for Case Series [12]. Non-randomized comparative studies of surgical or other interventional approaches were assessed using ROBINS-I (Risk Of Bias In Non-randomized Studies - of Interventions) [13]. For studies that could reasonably be classified as either observational cohorts or non-randomized intervention studies, the tool was selected according to the primary objective: ROBINS-I for intervention comparisons and NOS for exposure-outcome associations. 

Particular attention was given to confounding by indication in surgical comparisons because the selected repair approach could be influenced by RMT, niche morphology, symptoms, fertility goals, and clinician preference. No randomized controlled trials were included; therefore, the Cochrane RoB 2 tool was not applied. Each study was assessed according to the domains and appraisal criteria specified by the relevant tool, including selection and comparability for NOS-assessed studies; eligibility, exposure assessment, confounding, and outcome assessment for JBI analytical cross-sectional studies; case definition, case inclusion, clinical reporting, follow-up, and outcome reporting for JBI case series; and confounding, participant selection, intervention classification, deviations from intended interventions, missing data, outcome measurement, and selective reporting for ROBINS-I-assessed studies.

For presentation across appraisal tools, review-level summary judgments were expressed as low, moderate, or high concern, with "not applicable" used where relevant. JBI overall judgments reflected reviewer consensus on the number, severity, and likely impact of concerns; no numerical cutoff was applied. These summary labels were used to facilitate narrative comparison and did not replace the original tool-specific appraisal criteria. Disagreements in risk-of-bias judgments were resolved through discussion and consensus, with third-reviewer adjudication when required. Detailed domain-level assessments and overall judgments were presented separately for each appraisal tool. Risk-of-bias findings were used to guide interpretation of the evidence and were not used as post hoc exclusion criteria.

Data Synthesis

A structured narrative synthesis was performed because the included studies varied substantially in population, diagnostic criteria, intervention or exposure, comparator structure, outcome definition, analytical denominator, follow-up duration, and reporting format. A meta-analysis was not performed because sufficiently comparable effect estimates and consistently defined quantitative outcomes were unavailable across the included studies. Findings were organized into diagnostic and anatomical findings, fertility and reproductive outcomes, ART/IVF/ICSI outcomes, outcomes after surgical repair, and mechanistic or pathophysiological findings.

Study designs were not treated as equivalent sources of evidence. Comparative observational studies were given greater interpretive weight than uncontrolled case series, while mechanistic and descriptive studies were used primarily to contextualize potential biological or clinical pathways. Causal conclusions were interpreted cautiously because the evidence base consisted entirely of non-randomized studies. 

A formal GRADE (Grading of Recommendations Assessment, Development, and Evaluation) assessment was not undertaken. Given the broad review question, heterogeneous observational designs, variable outcome definitions and measurement approaches, and the absence of sufficiently comparable effect estimates for pooled analysis, certainty was considered narratively according to study design, sample size, risk of bias, consistency, directness, and outcome-measurement heterogeneity. A meta-analysis was not performed because the included studies differed substantially in their populations, diagnostic criteria, interventions, comparator structures, reproductive denominators, follow-up durations, and outcome definitions. Accordingly, the findings were synthesized narratively.

Results

Study Selection 

The combined PubMed/MEDLINE and Scopus searches identified 506 records. After removal of 71 duplicates, 435 unique database records remained, and supplementary searching added 11 records. In total, 446 records underwent title and abstract screening, of which 361 were excluded. A total of 85 reports were sought for retrieval, and 35 reports could not be retrieved. This represented 41.2% of the reports sought for retrieval. The 35 unavailable reports were identified in closed-access sources, and their full texts could not be accessed through the available institutional or library routes. They were documented separately in the screening records and were not included in the full-text eligibility assessment. Consequently, 50 full-text reports were assessed for eligibility. Fourteen full-text reports were excluded for the following reasons: no extractable fertility or reproductive outcomes or insufficient relevant data (n = 5); wrong population or exposure, or an exclusive focus on cesarean scar pregnancy without eligible fertility data (n = 3); review, protocol, non-original report, or another ineligible study design (n = 3); duplicate or overlapping data (n = 2); and a non-English full text that could not be reliably assessed (n = 1). Overall, 36 studies were included in the final narrative synthesis [14-49]. The study-selection process is summarized in Figure 1. 

**Figure 1:**
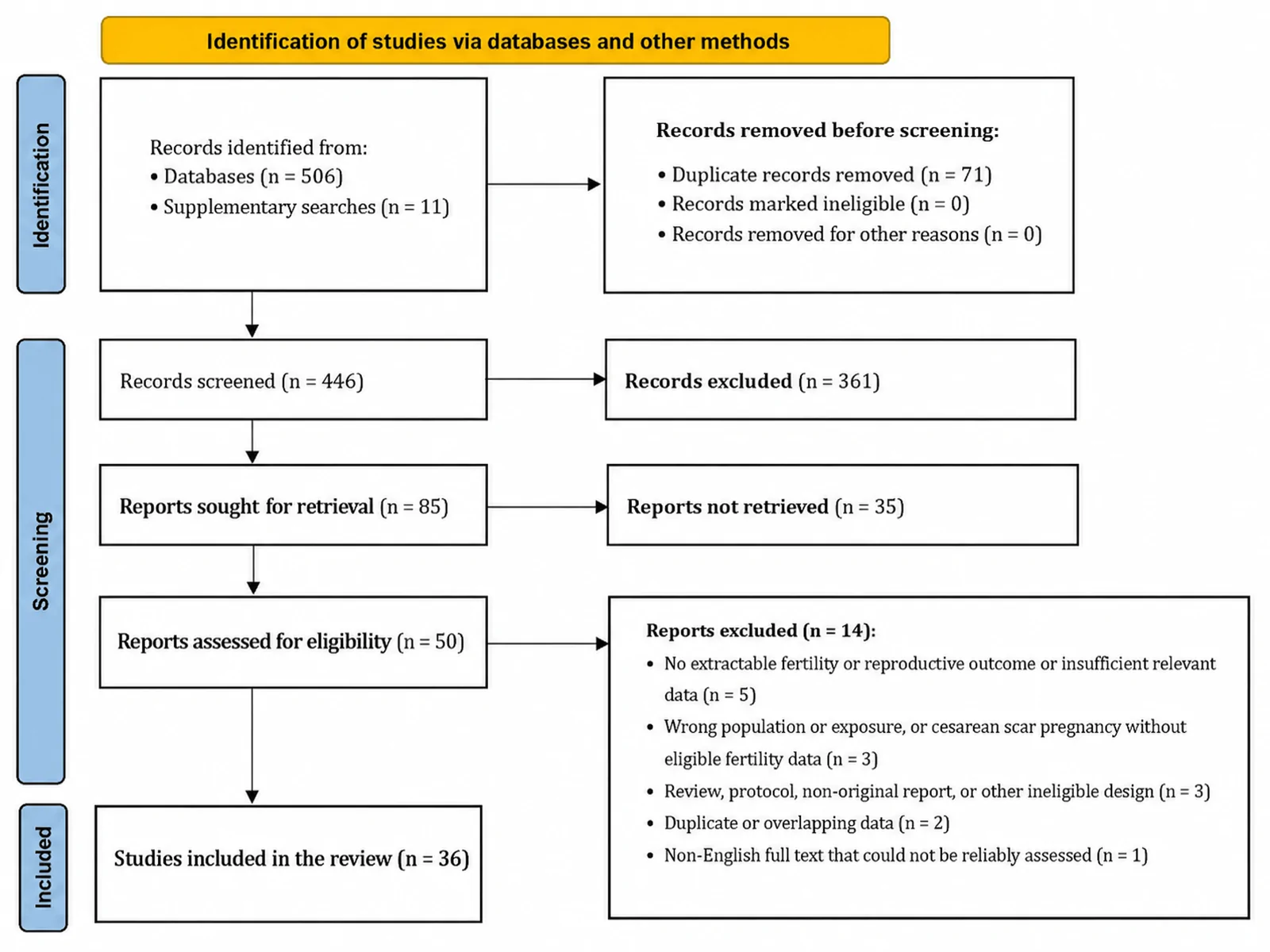
PRISMA 2020 flow diagram of study identification, screening, eligibility assessment, and inclusion PRISMA: Preferred Reporting Items for Systematic reviews and Meta-Analyses

Study Characteristics 

The 36 included studies were published between 2019 and 2026 and represented 15 countries [14-49]. However, the geographical distribution was uneven, with a substantial concentration of evidence from China and comparatively limited representation from Western countries, low-resource settings, and other regions. Additional contributions came from Europe, other parts of Asia, the Middle East, and the United States. Complete country-level details are provided in Table 3.

**Table 3:** Characteristics of the included studies ART: assisted reproductive technology; CE: chronic endometritis; CS: cesarean section; CSD: cesarean scar defect; CSS: cesarean scar syndrome; FET: frozen embryo transfer; HSG: hysterosalpingography; ICSI: intracytoplasmic sperm injection; IVF: in vitro fertilization; RMT: residual myometrial thickness; SI: secondary infertility; TVUS: transvaginal ultrasound

Study (Author, Year)	Country	Study Design	Population/Sample	Evidence Domain	Main Extracted Signal
Piriyev et al., 2022 [14]	Germany	Retrospective case series	28 women with current or potential fertility desire undergoing laparoscopic niche correction	Surgical repair	RMT increased from 2.0 to 8.7 mm; pregnancy occurred in 5/28 and live birth in 4/28.
Baldini et al., 2022 [15]	Italy	Retrospective case-control study	167 women with prior cesarean delivery undergoing IVF/ICSI	ART/embryo transfer	Day-5 ultrasound-guided transfer showed a lower cesarean scar pregnancy signal than day-3 transfer.
Nobuta et al., 2022 [16]	Japan	Ambidirectional cohort	Infertile women with and without cesarean scar syndrome; denominators varied by analysis	Mechanism	Chronic endometritis was more frequent in CSS, and TNF-alpha and IL-1beta levels were higher.
Nezhat et al., 2023 [17]	United States	Retrospective observational study	27 patients undergoing two-layer laparoscopic CSD repair	Surgical repair	Two-layer laparoscopic repair was followed by pregnancy in 73% of women desiring pregnancy.
Yang et al., 2023 [18]	China	Prospective observational comparative study	191 women undergoing hysteroscopic resection or vaginal repair for CSD	Surgical repair	In the fertility subgroup, pregnancy occurred in 69.2% after hysteroscopic resection and 58.3% after vaginal repair.
Coppenrath et al., 2024 [19]	Belgium	Retrospective case series	26 women with secondary infertility undergoing transvaginal niche repair	Surgical repair	RMT increased from 1.6 to 6.4 mm; pregnancy occurred in 64%.
Hosseini et al., 2024 [20]	Iran	Prospective cohort	46 patients with symptomatic isthmocele	Surgical repair	Laparoscopic and hysteroscopic repair were associated with improved anatomy and symptoms.
Cheng et al., 2025 [21]	China	Retrospective comparative cohort	154 women with non-severe CSD and RMT ≥3 mm	Surgical repair	Repregnancy occurred in 61.29% after hysteroscopic resection and 55.0% after laparoscopic repair.
Almasarweh et al., 2026 [22]	Germany	Retrospective observational study	51 women undergoing robotic-assisted laparoscopic niche repair	Surgical repair	RMT increased from 1.75 to 4.9 mm; pregnancy occurred in 26/36 and live birth in 17/36 among women with fertility follow-up.
Huang et al., 2026 [23]	China	Retrospective case series	26 women undergoing laparoscopic folding-suture repair	Surgical repair	RMT increased from 1.3 to 6.5 mm; pregnancy occurred in 12/16 women attempting conception.
Pabuccu, 2026 [24]	Turkey	Diagnostic case series	Seven women with prior cesarean delivery and secondary infertility	Diagnostic/anatomical	Hysterosalpingography pocket sign was described; all patients had secondary infertility and a TVUS-confirmed niche threshold of at least 2 mm.
Calzolari et al., 2019 [25]	Italy	Retrospective case series	35 consecutive women with symptomatic isthmocele	Surgical repair	Hysteroscopic isthmoplasty was followed by pregnancy in 9/16 women in the infertile subgroup.
Vegas Carrillo de Albornoz et al., 2019 [26]	Spain	Prospective case series	38 women with symptomatic isthmocele undergoing hysteroscopic correction	Surgical repair	Among women attempting conception, pregnancy occurred in 3/7 and live birth in 2/7.
Tsuji et al., 2020 [27]	Japan	Retrospective cohort	38 women with secondary infertility attributed to cesarean scar syndrome	Surgical repair	After hysteroscopic surgery, pregnancy occurred in 27/38 and live birth in 23/38; postoperative RMT was predictive.
Alalfy et al., 2020 [28]	Egypt	Prospective cross-sectional comparative study	110 women with secondary infertility, one prior cesarean delivery, and planned ICSI	Diagnostic/anatomical	Three-dimensional sonohysterography improved scar assessment compared with two-dimensional sonohysterography.
Szafarowska et al., 2021 [29]	Poland	Prospective comparative observational study	85 women with symptomatic isthmocele undergoing diagnostic or operative hysteroscopy	Surgical repair	Pregnancy occurred in 52.0% versus 26.3% across comparative management subgroups.
Zhang et al., 2021 [30]	China	Retrospective comparative cohort	76 women with CSD, RMT <3 mm, and fertility desire	Surgical repair	Pregnancy occurred in 50.0% after novel laparoscopic repair and 25.0% after traditional repair.
Karampelas et al., 2021 [31]	Belgium	Retrospective case series	31 women with RMT <5 mm and symptoms or secondary infertility	Surgical repair	In the infertility subgroup, pregnancy occurred in 10/12 after laparoscopic repair.
Bi et al., 2021 [32]	China	Retrospective comparative observational study	60 women with CSD plus benign surgical conditions and controls	Mechanism	Adhesion score was higher in CSD cases than controls, and inflammatory cytokines and tubal factors were assessed.
Zhang et al., 2022 [33]	China	Retrospective cohort	1,122 women younger than 40 years undergoing first FET after one previous delivery	ART/IVF	The CSD subgroup included 129 women; pregnancy and live-birth outcomes were compared by embryo-transfer strategy.
Alami et al., 2024 [34]	Afghanistan	Mixed retrospective-prospective observational study	9,207 women with previous cesarean delivery; symptomatic women aged 24-60 years were assessed	Prevalence/clinical profile	CSD prevalence was 0.62%, and secondary infertility was reported in 22% of CSD cases.
Chen et al., 2024 [35]	China	Comparative mechanistic case-control study	48 infertile women with CSD or infertile controls	Mechanism	A total of 162 bacterial and 42 fungal species differed according to CSD status.
Born et al., 2025 [36]	Germany	Retrospective case-control study	94 women with symptomatic or large CSD undergoing laparoscopic repair	Mechanism	Endometriosis was identified in 45/94; heavy menstrual bleeding was more frequent in women with uterine scar defects.
Sultan et al., 2025 [37]	Pakistan	Cross-sectional observational study	100 women aged 20-45 years with prior cesarean delivery, symptoms, and imaging-confirmed CSD	Diagnostic/anatomical	Mean defect depth was 3.4 ± 1.1 mm; intrauterine fluid was present in 29/39 infertile women.
Lawrenz et al., 2020 [38]	United Arab Emirates	Prospective observational exploratory study with retrospective FET comparison	103 women with secondary infertility, prior cesarean delivery, ultrasound-visible isthmocele, and IVF/ICSI stimulation	ART/IVF	Intracavitary fluid during ovarian stimulation and subsequent FET outcomes were assessed.
Ishikawa et al., 2023 [39]	Japan	Retrospective observational study	20 women with CSD, RMT ≤3 mm, secondary infertility, or concern regarding rupture risk undergoing abdominal repair	Surgical repair	Pregnancy occurred in 8/20, and live birth was reported in pregnancies reaching delivery.
Jordans et al., 2023 [40]	Netherlands	Prospective cohort	42 participants, including 31 women with niche and 11 controls	Mechanism	Ultrasound speckle tracking showed higher contraction amplitude in women with niche than controls.
Klein Meuleman et al., 2023 [41]	Netherlands	Prospective cohort	141 women with large niche, unexplained infertility, regular cycles, and RMT <3 mm or <50% of adjacent myometrium	Diagnostic/anatomical	Intrauterine fluid was present in 38/141; endometrial thickness did not differ significantly by fluid status.
Klein Meuleman et al., 2025 [42]	Netherlands	Prospective cohort	13 women with large niche, active wish to conceive, and scheduled laparoscopic repair	Mechanism	After repair, RMT increased from 0.4 to 6.2 mm and niche volume decreased.
Vissers et al., 2023 [43]	Netherlands	Prospective cohort	133 women with symptomatic large niche, RMT <3 mm, and pregnancy desire undergoing laparoscopic resection	Surgical repair	Ongoing pregnancy occurred in 83/133 and live birth in 77/133.
Wei et al., 2023 [44]	China	Retrospective propensity-score-matched cohort study	1,411 infertility patients undergoing hysteroscopy before matching	Mechanism	CSD was independently associated with chronic endometritis: adjusted OR 1.571, 95% CI 1.021-2.418.
Wu et al., 2025 [45]	China	Retrospective cohort	95 women with previous CSD undergoing hysteroscopic-assisted laparoscopic repair	Surgical repair	Pregnancy occurred in 16.7% versus 40.0% according to suturing technique.
Yao et al., 2023 [46]	China	Retrospective cohort with propensity-score matching	2,231 women with prior cesarean delivery undergoing IVF/ICSI embryo transfer	ART/IVF	The dataset included 2,515 cycles; niche was associated with lower pregnancy and live-birth outcomes.
Ahamed et al., 2023 [47]	United Arab Emirates	Retrospective case series	Five women aged 28-41 years with secondary infertility, CSD, and persistent hydrometra without hydrosalpinx	Surgical repair	Pregnancy occurred in 5/5 after repair, and live birth occurred in 4/5.
Halilzade and Seçen, 2025 [48]	Turkey	Retrospective observational cohort	41 women aged 18-50 years undergoing surgical repair for symptomatic isthmocele	Surgical repair	Among women desiring pregnancy, pregnancy occurred in 7/12 and live birth in 5/12.
Yin et al., 2026 [49]	China	Multicenter ambispective observational cohort	495 women with symptomatic CSD undergoing vaginal repair and subsequently attempting pregnancy	Surgical repair	Pregnancy occurred in 218/495 overall and in 83/141 women with prior secondary infertility.

The evidence base was entirely observational, and no randomized controlled trials were identified. The included designs comprised 15 cohort or comparative cohort studies [16,20,21,27,30,33,40-46,48,49], eight non-randomized surgical or clinical observational studies [17,18,22,29,32,34,38,39], eight case series [14,19,23-26,31,47], three case-control studies [15,35,36], and two cross-sectional studies [28,37].

Because the unit of analysis varied across studies and included individual participants, treatment cycles, and clinical records, a single combined sample size was not calculated. Of the 35 studies with a clearly reported denominator, 30 included fewer than 200 participants, cycles, or records [14,15,17-32,35-43,45,47,48]. One mechanistic study reported different denominators for separate analyses [16].

The included studies were grouped into five evidence domains. Surgical repair represented the largest domain (n = 20) and included hysteroscopic, laparoscopic, robotic-assisted laparoscopic, abdominal, transvaginal, and vaginal repair approaches [14,17-23,25-27,29-31,39,43,45,47-49]. Four studies directly evaluated assisted reproductive technology settings, including embryo-transfer timing, frozen embryo transfer after previous cesarean delivery, intracavitary fluid during ovarian stimulation, and uterine niche status during IVF or ICSI cycles [15,33,38,46].

Four studies focused primarily on diagnostic or anatomical assessment, including hysterosalpingography, two-dimensional (2D) and three-dimensional (3D) sonohysterography, niche depth, RMT, and intrauterine fluid [24,28,37,41]. One large observational study evaluated the prevalence and clinical profile of cesarean scar defects among women with previous cesarean delivery [34]. Seven studies examined mechanistic or pathophysiological pathways, which could be grouped broadly into inflammatory, anatomical or pelvic, microbial, and functional mechanisms [16,32,35,36,40,42,44].

Diagnostic methods varied according to the study objective and included transvaginal ultrasonography, saline infusion sonography or sonohysterography, hysteroscopy, hysterosalpingography, magnetic resonance imaging, and ultrasound speckle-tracking analysis. Definitions of cesarean scar defect were not uniform across studies. Several studies defined a niche using a depth threshold of at least 2 mm, whereas others focused on large defects characterized by RMT below 3 mm or less than 50% of the adjacent myometrial thickness [22,24,37,40,41]. The main characteristics of the included studies are summarized in Table 3. 

Diagnostic and Anatomical Findings

Diagnostic and anatomical studies focused mainly on niche depth, RMT, niche size, and intrauterine or intracavitary fluid, while postoperative studies also assessed restoration of the lower uterine segment. Across studies using laparoscopic, transvaginal, robotic-assisted, and vaginal repair, RMT generally increased after treatment [14,19,22,23,31,43,49]. Representative examples included an increase from 2.0 mm to 8.7 mm after laparoscopic repair [14], from 1.6 mm to 6.4 mm after transvaginal repair [19], and from 2.62 ± 1.14 mm to 7.18 ± 2.34 mm after vaginal repair [49].

Diagnostic studies provided complementary evidence for identifying fertility-relevant scar features. Alalfy et al. evaluated 110 women with secondary infertility planned for ICSI and supported 3D sonohysterography as a more detailed approach for scar measurement than two-dimensional assessment [28]. Pabuccu described the hysterosalpingography pocket sign in seven women with secondary infertility, suggesting that hysterosalpingography (HSG) may identify patients who require confirmatory scar assessment [24]. Sultan et al. reported a mean niche depth of 3.4 ± 1.1 mm and intrauterine fluid in 29 of 39 infertile women [37], whereas Klein Meuleman et al. found intrauterine fluid in 38 of 141 women with a large niche and unexplained infertility, although endometrial thickness did not differ significantly according to fluid status [41]. Overall, diagnostic assessment appeared most informative when scar morphology, RMT, fluid status, and reproductive context were considered together. The key diagnostic and anatomical findings are summarized in Table 4.

**Table 4:** Diagnostic and anatomical findings AUB: abnormal uterine bleeding; CSD: cesarean scar defect; HSG: hysterosalpingography; ICSI: intracytoplasmic sperm injection; MRI: magnetic resonance imaging; RMT: residual myometrial thickness; SHG: sonohysterography; SIS: saline infusion sonography; TVUS: transvaginal ultrasound

Study (Author, Year)	Assessment Approach	Key Anatomical Values	Fertility or Fluid Signal
Piriyev et al., 2022 [14]	TVUS, MRI, and hysteroscopy; large defects often defined by thin RMT	Mean RMT increased from 2.0 mm before repair to 8.7 mm after repair	Included women with fertility desire; one established secondary-infertility case was reported.
Yang et al., 2023 [18]	TVUS; CSD area categorized as ≤15, 15-25, or >25 mm²	Niche size was reported by area rather than separate depth and width	Postoperative fluid disappearance occurred in 70.1% after hysteroscopic resection and 60.2% after vaginal repair.
Coppenrath et al., 2024 [19]	Ultrasound-based niche assessment; very thin RMT selected for repair	Mean RMT increased from 1.6 mm to 6.4 mm	Pregnancy occurred in 64% after transvaginal repair.
Almasarweh et al., 2026 [22]	TVUS and hysterosalpingo-ultrasonography; niche depth threshold ≥2 mm	RMT increased from 1.75 ± 1.4 mm to 4.9 ± 3.4 mm; largest niche diameter decreased from 11.3 ± 5.0 mm to 9.1 ± 4.7 mm	Pregnancy occurred in 72.2% of women with fertility follow-up.
Pabuccu, 2026 [24]	HSG, TVUS, and hysteroscopy; HSG pocket sign with TVUS confirmation	TVUS niche threshold was ≥2 mm	All seven patients had secondary infertility; postmenstrual spotting was present in 5/7.
Alalfy et al., 2020 [28]	Two-dimensional and three-dimensional SHG	RMT, depth, and width were measured by both methods	Study population consisted entirely of women with secondary infertility planned for ICSI.
Alami et al., 2024 [34]	TVUS with descriptive assessment of pouch-like scar defect	RMT was measured prospectively in CSD cases	Secondary infertility was reported in 22% of CSD cases; AUB, dysmenorrhea, and pelvic pain were common symptoms.
Sultan et al., 2025 [37]	TVUS, SIS, and hysteroscopy; defect >3 mm considered clinically significant	Mean niche depth was 3.4 ± 1.1 mm	Intrauterine fluid was present in 29/39 infertile women; niche depth >3 mm was associated with infertility.
Jordans et al., 2023 [40]	TVUS speckle tracking; niche indentation ≥2 mm	Median RMT was 1.3 mm in the niche group versus 10.8 mm in controls; median niche depth was 6.5 mm	Subfertility was present in 18/31 women with niche; higher contraction amplitude was proposed as an implantation-related mechanism.
Klein Meuleman et al., 2023 [41]	TVUS; large niche defined as RMT <3 mm or <50% of adjacent myometrium	RMT was 1.0 mm without intrauterine fluid and 1.5 mm with intrauterine fluid	Intrauterine fluid was present in 38/141 women; all participants had unexplained infertility.
Klein Meuleman et al., 2025 [42]	TVUS speckle tracking before and after laparoscopic repair	RMT increased from 0.4 ± 0.5 mm to 6.2 ± 1.5 mm; depth, width, and length all decreased after repair	Fluid resolved from 3/13 before repair to 0/13 after repair; 12/13 women had infertility.

Fertility, Reproductive, Surgical, and ART Outcomes

Reproductive outcomes were reported most often after surgical repair, although denominators varied across studies. Overall, hysteroscopic and hysteroscopy-based studies showed pregnancies after treatment in selected women with infertility or fertility desire. Study-specific findings included pregnancy in nine of 16 infertile women after hysteroscopic isthmoplasty in Calzolari et al.'s study [25], three of seven women attempting conception after hysteroscopic correction in the study by Vegas Carrillo de Albornoz et al. [26], and 27 of 38 women after hysteroscopic surgery for cesarean scar syndrome in the study by Tsuji et al. [27]. Comparative studies reported pregnancy in 27 of 39 women after hysteroscopic resection versus 14 of 24 after vaginal repair in Yang et al.'s study [18], and in 13 of 25 after operative hysteroscopy versus five of 19 after diagnostic hysteroscopy in the study by Szafarowska et al. [29]. These findings suggest a reproductive signal after hysteroscopic management in selected patients, but non-randomized designs, anatomy-based procedure selection, and confounding by indication limit direct comparisons between approaches. Patients with thinner residual myometrium or more severe defect morphology may preferentially undergo reconstructive procedures, whereas patients with less severe defects may undergo hysteroscopic treatment; observed differences in pregnancy rates may therefore reflect baseline case selection rather than differences in treatment efficacy.

Laparoscopic, robotic-assisted, abdominal, transvaginal, and vaginal reconstructive studies likewise linked anatomical restoration with subsequent fertility follow-up. Pregnancy was reported in 10 of 12 women in the infertility subgroup of the study by Karampelas et al. [31], 26 of 36 women with fertility follow-up after robotic-assisted laparoscopic repair in Almasarweh et al.'s study [22], 12 of 16 women desiring fertility after laparoscopic folding suture in Huang et al.'s study [23], eight of 20 women after abdominal repair in Ishikawa et al.'s study [39], and 218 of 495 women attempting pregnancy after vaginal repair in Yin et al.'s study [49]. Live-birth reporting was less consistent, but live births were documented after several reconstructive approaches, including hysteroscopic, laparoscopic, robotic-assisted, and vaginal repair [22,27,30,43,49].

ART-focused evidence suggested that CSD may influence reproductive outcomes through embryo-transfer, fluid-related, and implantation-related pathways. Regarding embryo-transfer timing, Baldini et al. reported cesarean scar pregnancy in two of 22 day-3 embryo transfers and in none of 76 day-5 transfers among women with isthmocele [15]. In relation to intracavitary fluid, Lawrenz et al. found that fluid developed in approximately 40% of women with isthmocele during ovarian stimulation, while frozen embryo-transfer outcomes were similar to controls when fluid was absent before transfer [38]. For broader reproductive outcomes, Zhang et al. reported lower clinical pregnancy and live-birth outcomes after frozen-thawed embryo transfer among women with CSD [33], and Yao W et al. reported lower implantation, clinical pregnancy, and live-birth outcomes together with higher early miscarriage among women with uterine niche [46]. The main reproductive, surgical, and ART outcomes are summarized in Table 5.

**Table 5:** Fertility, reproductive, surgical, and ART outcomes ART: assisted reproductive technology; CSD: cesarean scar defect; CSS: cesarean scar syndrome; FET: frozen embryo transfer; ICSI: intracytoplasmic sperm injection; IVF: in vitro fertilization; RMT: residual myometrial thickness

Study (Author, Year)	Setting and Denominator	Repair or ART Context	Key Reproductive Outcomes	Birth, Loss, or Complication Signal
Piriyev et al., 2022 [14]	28 women undergoing laparoscopic repair	Laparoscopic niche excision/repair with hysteroscopic guidance	Pregnancy occurred in 5/28	Live birth occurred in 4/28; one miscarriage was reported.
Baldini et al., 2022 [15]	98 women with isthmocele and 65 comparators	IVF/ICSI with embryo-transfer timing comparison	Clinical pregnancy occurred in 8/22 day-3 transfers and 30/76 day-5 transfers	Cesarean scar pregnancy occurred in 2/22 day-3 transfers and 0/76 day-5 transfers.
Nezhat et al., 2023 [17]	27 patients undergoing two-layer laparoscopic repair	Laparoscopic CSD repair	Pregnancy occurred in about 73% of women desiring pregnancy	No uterine rupture or cesarean scar pregnancy was reported.
Yang et al., 2023 [18]	191 women; fertility subgroup of 63	Hysteroscopic resection versus vaginal repair	Pregnancy occurred in 27/39 after hysteroscopic resection and 14/24 after vaginal repair	Live birth occurred in 24/39 and 9/24, respectively; miscarriage occurred in 2/27 and 4/14 pregnancies.
Coppenrath et al., 2024 [19]	26 women with secondary infertility	Transvaginal niche repair	Pregnancy occurred in 64%	No major obstetric complications were reported.
Cheng et al., 2025 [21]	154 women with non-severe CSD and RMT ≥3 mm	Hysteroscopic resection versus laparoscopic defect repair	Repregnancy occurred in 61.29% after hysteroscopic resection and 55.0% after laparoscopic repair	No placenta implantation abnormality or uterine rupture was reported.
Almasarweh et al., 2026 [22]	51 women; fertility follow-up in 36	Robotic-assisted laparoscopic niche resection and double-layer repair	Pregnancy occurred in 26/36	Live birth occurred in 17/36; miscarriage occurred in 5/26 pregnancies.
Huang et al., 2026 [23]	26 women; 16 attempting conception	Laparoscopic folding-suture repair	Pregnancy occurred in 12/16	Pregnancy-loss and live-birth outcomes were not fully reported
Calzolari et al., 2019 [25]	35 women; infertile subgroup of 16	Hysteroscopic isthmoplasty	Pregnancy occurred in 9/16 infertile women	Live-birth and pregnancy-loss outcomes were not separately reported.
Vegas Carrillo de Albornoz et al., 2019 [26]	38 women; seven attempting conception	Hysteroscopic correction	Pregnancy occurred in 3/7	Live birth occurred in 2/7; one miscarriage occurred among three pregnancies.
Tsuji et al., 2020 [27]	38 women with secondary infertility attributed to CSS	Hysteroscopic surgery	Pregnancy occurred in 27/38	Live birth occurred in 23/38; miscarriage occurred in 3/27 pregnancies.
Szafarowska et al., 2021 [29]	85 women; fertility subgroup of 44	Operative versus diagnostic hysteroscopy	Pregnancy occurred in 13/25 after operative hysteroscopy and 5/19 after diagnostic hysteroscopy	Most reported pregnancies were spontaneous.
Zhang et al., 2021 [30]	76 women with CSD, RMT <3 mm, and fertility desire	Novel laparoscopic repair versus traditional repair	Pregnancy occurred in 20/40 versus 9/36	Live birth occurred in 18/40 versus 8/36; one cesarean scar pregnancy occurred in the traditional-repair group.
Karampelas et al., 2021 [31]	31 women; infertility subgroup of 12	Laparoscopic excision and layered repair	Pregnancy occurred in 10/12	At least seven live births were reported; no uterine rupture was reported.
Zhang et al., 2022 [33]	1,122 women undergoing first FET; 129 with CSD	Frozen-thawed embryo transfer after prior delivery	CSD was associated with lower clinical pregnancy in adjusted analyses	Live-birth outcomes were lower in the CSD subgroup.
Lawrenz et al., 2020 [38]	103 women with isthmocele during stimulation; FET comparison included 53 cases and 442 controls	IVF/ICSI stimulation followed by FET	Intracavitary fluid developed in approximately 40% during ovarian stimulation	When fluid was absent before transfer, FET outcomes were similar to controls.
Ishikawa et al., 2023 [39]	20 women undergoing abdominal repair	Open abdominal CSD excision and multilayer repair	Pregnancy occurred in 8/20	Live birth was achieved in pregnancies reaching delivery; one missed abortion was reported.
Vissers et al., 2023 [43]	133 women desiring pregnancy	Laparoscopic niche resection and repair	Ongoing pregnancy occurred in 83/133	Live birth occurred in 77/133; miscarriage occurred in 11/133.
Wu et al., 2025 [45]	95 women; fertility-requirement subgroup assessed	Hysteroscopy-assisted laparoscopic repair; incise-and-suture versus fold-and-suture	Pregnancy occurred in 5/30 versus 10/25	Early delivery data were incomplete at report time, but miscarriage was uncommon.
Yao et al., 2023 [46]	2,515 IVF/ICSI cycles, including 179 niche cycles	IVF/ICSI embryo transfer with propensity-score matching	Niche was associated with lower implantation and clinical pregnancy	Live birth was lower and early miscarriage was higher in the niche group.
Ahamed et al., 2023 [47]	Five women with CSD, persistent hydrometra, and secondary infertility	Hysteroscopic and/or laparoscopic repair followed by fertility treatment	Pregnancy occurred in 5/5	Live birth occurred in 4/5; one miscarriage occurred at 10 weeks.
Halilzade and Secen, 2025 [48]	41 women; fertility-desire subgroup of 12	Hysteroscopic versus abdominal repair	Pregnancy occurred in 7/12	Live birth occurred in 5/12; mean time to conception was 8.4 ± 4.4 months.
Yin et al., 2026 [49]	495 women attempting pregnancy after vaginal repair	Vaginal excision of niche and multilayer uterine repair	Pregnancy occurred in 218/495 overall and 83/141 with prior secondary infertility	Live birth occurred in 175/232 pregnancies; miscarriage occurred in 42/232 pregnancies.

Mechanistic and Pathophysiological Findings

Inflammatory findings provided the clearest initial mechanistic signal linking CSD with impaired fertility. Nobuta et al. reported chronic endometritis in 65.8% of infertile women with cesarean scar syndrome compared with 46.0% of infertile comparators, together with higher tumor necrosis factor-alpha and interleukin-1 beta levels [16]. Wei et al. similarly reported a higher prevalence of chronic endometritis among women with CSD [44]. These findings support chronic endometrial inflammation as one plausible pathway through which the defect may affect reproductive function.

Other studies extended the mechanistic model beyond inflammation. Chen et al. found marked bacterial and fungal differences between infertile women with CSD and infertile controls, supporting a possible microbiome-related pathway [35]. Born et al. identified histologically confirmed endometriosis in nearly half of women undergoing laparoscopic CSD repair, indicating that coexisting endometriosis may contribute to symptoms or fertility impairment in some patients [36]. Contractility studies also suggested a functional mechanism: Jordans et al. reported higher subendometrial contraction amplitude in women with a niche than in controls [40], while Klein Meuleman et al. (2025) found that laparoscopic repair increased RMT, reduced niche volume, resolved intrauterine fluid, and shifted contractility toward a more normal pattern [42]. Overall, mechanistic studies implicated multiple potential pathways, including scar anatomy, retained fluid, chronic inflammation, dysbiosis, endometriosis, pelvic adhesions, tubal factors, and altered uterine peristalsis. Mechanistic findings are summarized in Table 6.

**Table 6:** Mechanistic and pathophysiological findings CE: chronic endometritis; CI: confidence interval; CSD: cesarean scar defect; CSS: cesarean scar syndrome; HMB: heavy menstrual bleeding; IUF: intrauterine fluid; OR: odds ratio; RMT: residual myometrial thickness; TNF: tumor necrosis factor

Study (Author, Year)	Mechanistic Focus	Sample/Comparator	Key Numerical Findings	Fertility Relevance
Nobuta et al., 2022 [16]	Chronic endometritis and uterine inflammatory cytokines	Infertile women with CSS versus infertile women without CSS	Chronic endometritis: 65.8% versus 46.0%; TNF-alpha and IL-1 beta were significantly higher in CSS	Supports chronic endometrial inflammation as a potential fertility-impairing pathway
Bi et al., 2021 [32]	Pelvic inflammation, adhesions, and tubal patency	30 women with CSD versus 30 surgical controls	Adhesion score: 4.67 ± 0.90 versus 0.47 ± 0.90; 21/30 tubes unobstructed; 12/30 repregnant	Suggests CSD may coexist with pelvic inflammatory and tubal factors
Chen et al., 2024 [35]	Microbiome, mycobiome, and metabolite interactions	24 infertile women with CSD versus 24 infertile controls	162 bacterial species and 42 fungal species differed by CSD status	Supports a possible dysbiosis-related mechanism in infertile women with CSD
Born et al., 2025 [36]	Retrograde menstruation and extragenital endometriosis	94 women with symptomatic or large CSD undergoing laparoscopic repair	Endometriosis: 45/94; heavy menstrual bleeding: 38% versus 8.3%; retroflexion: 57% versus 35%	Indicates that coexisting endometriosis may contribute to reproductive impairment in some patients
Jordans et al., 2023 [40]	Subendometrial peristalsis and contractility	31 women with niche versus 11 controls without prior cesarean scar	Overall contraction amplitude: 8.5 ± 4.2% versus 2.9 ± 1.3%	Suggests altered uterine contractility as a potential implantation-related mechanism
Klein Meuleman et al., 2023 [41]	Intrauterine fluid and endometrial development	141 infertile women with large niche; 38 with IUF versus 103 without IUF	Endometrial thickness: 6.0 mm with IUF versus 6.6 mm without IUF; no significant difference	Shows that IUF may be clinically relevant even when endometrial thickness is not reduced
Klein Meuleman et al., 2025 [42]	Reversibility of abnormal peristalsis after repair	13 women with large niche and pregnancy wish; within-person preoperative versus postoperative comparison	RMT increased by 5.8 mm; niche volume decreased by 963.9 mm³; IUF resolved from 3/13 to 0/13	Supports anatomical and functional improvement after repair
Wei et al., 2023 [44]	Chronic endometritis susceptibility	170 women with CSD versus 331 propensity-matched controls	Chronic endometritis: 49/170 versus 68/331; adjusted OR 1.571, 95% CI 1.021-2.418	Supports an independent association between CSD and chronic endometritis

Risk of Bias

Risk of bias was assessed according to study design and was used to guide interpretation rather than to determine eligibility. Overall, the most frequent limitations were retrospective design, small fertility subgroups, non-randomized procedure selection, confounding by indication, incomplete reproductive denominators, variable follow-up duration, and inconsistent reporting of pregnancy-attempt status. Cross-sectional and diagnostic studies generally had clearer eligibility and measurement procedures, whereas comparative surgical and ART studies were more vulnerable to selection bias and confounding because the treatment route, embryo-transfer strategy, or repair technique was often determined by niche anatomy, RMT, symptoms, fertility desire, or clinician preference. For clarity, risk-of-bias findings are presented by assessment tool and study design. Risk-of-bias judgments are presented as low risk, moderate risk, high risk, and not applicable.

The NOS was used for observational cohort, case-control, and non-interventional comparative studies (Table 7). Most NOS-assessed studies had moderate overall concern. Yao et al.'s study [46] had lower concern across selection, comparability, and outcome domains. High overall concern was mainly driven by limited comparability, incomplete adjustment for confounding, and small or selected comparator groups, particularly in studies where fertility outcomes may have been influenced by baseline reproductive status, embryo-transfer strategy, symptoms, or underlying pelvic factors.

**Table 7:** Risk-of-bias assessment using the Newcastle-Ottawa scale (NOS) L: low risk; M: moderate risk; H: high risk

Study (Author, Year)	Selection	Comparability	Outcome	Overall
Baldini et al., 2022 [15]	M	H	M	H
Nobuta et al., 2022 [16]	M	M	M	M
Bi et al., 2021 [32]	H	H	M	H
Zhang et al., 2022 [33]	L	M	L	M
Chen et al., 2024 [35]	M	M	L	M
Born et al., 2025 [36]	M	M	L	M
Lawrenz et al., 2020 [38]	M	M	M	M
Jordans et al., 2023 [40]	M	M	L	M
Klein Meuleman et al., 2023 [41]	M	M	L	M
Wei et al., 2023 [44]	M	L	L	M
Yao et al., 2023 [46]	L	L	L	L

The JBI Analytical Cross-Sectional Checklist was used for cross-sectional diagnostic or prevalence studies. The JBI analytical cross-sectional risk-of-bias summary is presented in Table 8. Cross-sectional evidence was useful for diagnostic and prevalence description but remained limited for causal inference. Alalfy et al.'s study [28] was judged to have low concern because the eligible population was clearly defined, imaging assessment was standardized, and the relevant diagnostic measurements were reported adequately. The study by Sultan et al. [37] had moderate concern because the population and diagnostic assessment were reasonably described, but control of confounding and statistical adjustment were limited. Alami et al.'s study [34] had high concern because the sampling approach, comparability, and control of potential confounding were less robust, making prevalence and symptom associations more vulnerable to selection and measurement bias.

**Table 8:** Risk-of-bias assessment using the JBI analytical cross-sectional checklist L: low risk; M: moderate risk; H: high risk; N/A: not applicable; JBI: Joanna Briggs Institute (formerly)

Study (Author, Year)	Eligibility/setting	Exposure	Confounding/control	Outcomes/statistics	Overall
Alalfy et al., 2020 [28]	L	L	N/A	L	L
Sultan et al., 2025 [37]	L	L	M	M	M
Alami et al., 2024 [34]	M	M	H	M	H

The JBI Case-Series Checklist was used for single-arm case series and surgical follow-up cohorts (Table 9). Among the 15 studies assessed using the JBI Case Series Checklist, 10 had moderate overall risk of bias, two had low risk, and three had high risk. The main concerns were incomplete or unclear consecutive case inclusion, selection of analysis sets based on follow-up or image quality, variable completeness or duration of follow-up, and limited analytical methods in small uncontrolled studies. The studies by Coppenrath et al. [19] and Calzolari et al. [25] had the lowest overall concern. Higher overall concern was assigned to the stuy by Piriyev et al. [14], Klein Meuleman et al. [42], and Yin et al. [49], mainly because of follow-up-dependent inclusion, exclusion of a large proportion of potentially eligible participants, or substantial loss to follow-up and restriction of the final analysis population.

**Table 9:** Risk-of-bias assessment using the JBI case series checklist L: low risk; M: moderate risk; H: high risk; JBI: Joanna Briggs Institute (formerly)

Study (Author, Year)	Eligibility	Condition Assessment	Case Inclusion	Clinical Reporting	Follow-up	Analysis	Overall
Piriyev et al., 2022 [14]	L	L	H	L	H	M	H
Nezhat et al., 2023 [17]	M	M	M	L	M	M	M
Coppenrath et al., 2024 [19]	L	L	L	L	L	L	L
Almasarweh et al., 2026 [22]	L	L	L	L	M	M	M
Huang et al., 2026 [23]	L	L	M	L	M	L	M
Pabuccu, 2026 [24]	M	L	M	M	L	L	M
Calzolari et al., 2019 [25]	L	L	L	L	L	L	L
Vegas Carrillo de Albornoz et al., 2019 [26]	L	L	L	L	M	M	M
Tsuji et al., 2020 [27]	L	L	L	L	M	L	M
Karampelas et al., 2021 [31]	L	M	M	L	M	L	M
Ishikawa et al., 2023 [39]	L	L	M	L	M	L	M
Klein Meuleman et al., 2025 [42]	L	L	H	L	L	M	H
Vissers et al., 2023 [43]	L	L	L	L	M	L	M
Ahamed et al., 2023 [47]	L	L	M	L	M	M	M
Yin et al., 2026 [49]	L	L	H	L	M	M	H

ROBINS-I was used for non-randomized comparative intervention studies. The ROBINS-I risk-of-bias summary is presented in Table 10. ROBINS-I assessments showed the highest concern among comparative surgical studies. The major limitation was confounding by indication: patients were not randomized to hysteroscopic, laparoscopic, vaginal, abdominal, or combined repair, and treatment selection was often influenced by RMT, niche size, symptoms, fertility desire, surgeon preference, or clinical severity. As a result, differences in pregnancy or live-birth outcomes between surgical approaches should not be interpreted as direct evidence that one technique is superior. These studies are best interpreted as supportive evidence for anatomy-guided management rather than definitive comparative effectiveness evidence. Accordingly, pregnancy and live-birth rates should be interpreted as study-specific outcomes in selected populations rather than as comparative estimates applicable across all surgical techniques.

**Table 10:** Risk-of-bias assessment using the ROBINS-I tool L: low risk; M: moderate risk; H: high risk; ROBINS-I: Risk Of Bias In Non-randomized Studies of Interventions

Study (Author, Year)	Confounding	Selection	Classification/Deviations	Missing Data	Measurement	Reporting	Overall
Yang et al., 2023 [18]	H	M	L	M	L	M	H
Hosseini et al., 2024 [20]	H	H	L	M	H	M	H
Cheng et al., 2025 [21]	H	M	L	M	M	M	H
Szafarowska et al., 2021 [29]	H	H	L	M	M	M	H
Zhang et al., 2021 [30]	M	M	L	L	L	M	M
Wu et al., 2025 [45]	H	H	L	M	M	M	H
Halilzade I, Islek Secen, 2025 [48]	H	H	L	M	L	M	H

Overall, the risk-of-bias assessment supports cautious interpretation. The evidence base provides clinically important signals linking CSD/isthmocele with infertility, ART outcomes, chronic inflammation, intrauterine fluid, altered contractility, and reproductive outcomes after repair. However, most evidence remains observational, and many fertility outcomes are vulnerable to selection bias, confounding, small sample size, and inconsistent denominator reporting. Therefore, risk of bias was incorporated into the narrative synthesis and phrased cautiously rather than as definitive causal claims.

Discussion

The findings of this systematic review suggest that CSD/isthmocele may be associated with secondary infertility and adverse reproductive outcomes in selected women after cesarean delivery. Across the evidence base, the clinically important issue was not simply whether a niche was present, but whether it formed part of a fertility-relevant phenotype characterized by thin residual myometrium, intrauterine or intracavitary fluid, inflammatory changes, altered uterine contractility, or unfavorable conditions during assisted reproduction. This distinction is important in practice because many niches may be incidental, whereas others have anatomical or functional features that warrant closer fertility-focused assessment.

Diagnostic and Anatomical Interpretation

The diagnostic evidence indicates that CSD should not be interpreted as a simple present-or-absent finding. Fertility-relevant assessment appears to depend on the combination of RMT, niche size, fluid retention, associated symptoms, and, where relevant, functional imaging findings. Studies using sonohysterography, hysterosalpingography, transvaginal ultrasound, hysteroscopy, and speckle-tracking ultrasound showed substantial variation in scar morphology and function [24,28,37,40-42]. For clinicians, a large or thin-walled niche accompanied by intracavitary fluid, bleeding symptoms, or abnormal uterine dynamics is therefore more informative than an incidental small defect seen on imaging alone.

Reproductive and Surgical Interpretation

The reproductive evidence supports an association between CSD/isthmocele and impaired fertility, but it does not establish causation. Pregnancy and live-birth outcomes have been reported after hysteroscopic and reconstructive repair in selected women [14,17-23,25-27,29-31,39,43,45,47-49], but these studies used different patient-selection criteria, anatomical thresholds, surgical techniques, follow-up periods, and reproductive endpoints. Importantly, surgical treatment was not randomly assigned. Patients with very thin RMT, deeper defects, or unfavourable niche morphology may be preferentially directed toward laparoscopic, abdominal, robotic, transvaginal, or vaginal reconstruction, whereas patients with thicker residual myometrium may be more likely to undergo hysteroscopic treatment. This confounding by indication makes direct comparison of postoperative pregnancy rates unreliable. Therefore, a higher or lower pregnancy rate reported for one approach cannot be interpreted as proof of clinical superiority. The available evidence supports individualized, anatomy-guided treatment selection rather than a universal procedural hierarchy. Hysteroscopic treatment may be considered in selected less severe defects with adequate myometrial thickness, whereas reconstructive approaches may be considered when residual myometrium is thin or restoration of myometrial integrity is a major objective.

Assisted Reproduction and Implantation-Related Findings

ART-focused studies suggest that CSD may influence implantation through a combination of scar anatomy and the uterine environment [15,33,38,46]. Intracavitary fluid deserves particular attention because its presence or persistence before embryo transfer may be more clinically relevant than niche detection alone. In the available cohorts, fluid status around transfer, embryo-transfer strategy, comparator selection, and adjustment for confounders all influenced reported outcomes. Thus, intracavitary fluid may be a useful feature to consider when interpreting ART outcomes in women with CSD, but the current evidence is not sufficiently uniform to define a standard transfer or treatment strategy.

Mechanistic Interpretation

The mechanistic findings support a multifactorial model, but they should not be generalized to every woman with CSD. Evidence for chronic endometritis, inflammatory cytokine changes, or other inflammatory abnormalities came from selected studies and patient subsets rather than from biopsy or inflammatory-marker assessment across the entire evidence base [16,32,44]. Other studies suggested possible contributions from dysbiosis, coexisting endometriosis, pelvic or tubal factors, and altered subendometrial contractility [35,36,40,42]. These findings make biological sense when considered alongside retained fluid and scar anatomy, but they remain plausible mechanisms rather than universal features or proven causal pathways.

Strength of the Evidence

A strength of the evidence is that several study types point toward a similar clinical concept: some cesarean scar niches appear to have anatomical, functional, or reproductive relevance [14-49]. However, the strength of that convergence should not be overstated. The evidence is almost entirely observational and is affected by small or single-center cohorts, inconsistent definitions, heterogeneous comparators, variable follow-up, non-uniform study eligibility criteria, and different fertility outcomes. These differences reduce the precision of the overall inference and explain why narrative synthesis was more appropriate than quantitative pooling.

Clinical and Research Implications

The geographical concentration of the evidence may limit the generalizability of the findings. A substantial proportion of the included studies originated from China, where cesarean-delivery patterns, referral pathways, diagnostic practices, reproductive-health services, and access to specialized scar-repair surgery may differ from those in Western countries and low-resource settings. National guidelines and local diagnostic thresholds may influence which niches are considered clinically significant and which patients are referred for surgical treatment. Differences in healthcare access, IVF funding structures, insurance coverage, and availability of reproductive surgery may also affect patient selection and the likelihood of receiving assisted reproductive treatment. In addition, embryo-transfer practices and clinical decision-making may vary between healthcare systems, potentially influencing implantation, pregnancy, and live-birth outcomes. The Afghan cohort provides some representation from a lower-resource setting [34]; however, a single study cannot capture the diversity of reproductive-care pathways in low- and middle-income countries. Therefore, the findings should be applied cautiously outside the settings in which the primary evidence was generated.

Clinically, the findings support individualized assessment of women with secondary infertility after cesarean delivery rather than routine intervention for every detected niche. A higher-risk fertility-related phenotype may be suggested by combinations of postmenstrual spotting or pelvic symptoms, thin residual myometrium, intracavitary fluid, recurrent implantation problems, or other adverse anatomical or functional features. In contrast, an asymptomatic small niche without these features may have less immediate reproductive significance. The current evidence does not support routine repair of all CSDs; management should be guided by symptoms, reproductive goals, scar anatomy, fluid status, and the broader infertility assessment.

Future research should use standardized CSD definitions and patient-selection criteria, clearly separate confirmed secondary infertility from general fertility desire, and report RMT, niche dimensions, fluid status, and spontaneous versus ART-mediated conception consistently. Larger prospective comparative studies, and controlled trials where feasible, are needed to clarify which patients benefit from intervention and whether particular treatment approaches improve reproductive outcomes beyond the effects of baseline anatomy and other causes of infertility.

Limitations

This review has several limitations. The protocol was not prospectively registered in PROSPERO, OSF, or another systematic-review registry, which limits independent external verification of prespecified methods and outcomes. However, no deviations occurred between the initial review design and the final study selection, data extraction, risk-of-bias assessment, outcome interpretation, or narrative synthesis.

The formal bibliographic search was limited to PubMed/MEDLINE and Scopus and did not include Embase or the Cochrane Central Register of Controlled Trials. This limitation may have reduced the comprehensiveness of the evidence base. However, the search was supplemented by Google Scholar, PubMed Central, publisher webpages, DOI records, citation tracking, and manual reference-list screening, and only those studies that were indexed only in Embase, Cochrane Central Register of Controlled Trials (CENTRAL), or other databases may have been missed, including some European publications, conference records, and clinical-trial-related evidence.

Only completed English-language full-text reports with sufficient extractable data were included. Of the 85 reports sought for retrieval, 35 could not be obtained, corresponding to 41.2%. Although the reports were recorded as not retrieved and retrieval procedures were undertaken, their absence may have introduced availability or retrieval bias. Inaccessible reports may have differed from the included studies in publication year, setting, study quality, sample size, or direction of findings. These restrictions mean that relevant evidence may have been omitted and reduce confidence that the review captured the full range of published findings.

The main limitation was substantial clinical and methodological heterogeneity. Included studies differed in their populations, inclusion and exclusion criteria, CSD definitions, anatomical thresholds, surgical or ART protocols, comparators, outcome definitions, analytical denominators, and follow-up periods. This variability precluded meaningful quantitative pooling and prevents the current evidence from defining a single standardized diagnostic or treatment pathway. Many studies were retrospective, non-randomized, single-center, or small, leaving the evidence vulnerable to selection bias, confounding by indication, and imprecision. Fertility and reproductive outcomes were also reported inconsistently, and publication bias or selective outcome reporting cannot be excluded. Taken together with the unretrieved reports and heterogeneity described above, these limitations mean that the overall findings should be interpreted as clinically suggestive rather than definitive comparative or causal evidence. 

## Conclusions

CSD/isthmocele may be associated with secondary infertility and adverse reproductive outcomes, particularly when accompanied by thin residual myometrium, intracavitary fluid, inflammatory changes, altered uterine contractility, or other unfavorable implantation-related features. Its reproductive significance should be assessed using scar morphology, residual myometrial thickness, fluid status, symptoms, and the broader infertility context rather than niche presence alone. Although surgical repair has been associated with anatomical improvement and subsequent pregnancies or live births in selected patients, the evidence remains observational and heterogeneous, with no single treatment approach shown to be universally superior. Therefore, not every detected isthmocele should be considered a cause of infertility or an automatic indication for surgery. Prospective comparative studies using standardized diagnostic criteria and clearly defined fertility and reproductive outcomes are needed to identify patients most likely to benefit from intervention.
